# Two primary melanoma in situ lesions of the vulva: Treatment with imiquimod

**DOI:** 10.1016/j.jdcr.2026.02.021

**Published:** 2026-02-17

**Authors:** Bianca N. Coffel, Kelly H. Hall, David W.L. Cowart

**Affiliations:** a Department of Dermatology, San Antonio Uniformed Services Health Education Consortium, Lackland AFB, Texas; b Department of Pathology, Brooke Army Medical Center, Fort Sam Houston, San Antonio, Texas

**Keywords:** melanoma in situ, topical imiquimod, vulvar malignancy

## Introduction

We present a case of a 70-year-old female diagnosed with melanoma in situ (MIS) of the left labia minora 6 years after a diagnosis of MIS of the right labia majora that was treated with Modified (Slow) Mohs Micrographic Surgery (MMS). Our patient was started on a course of topical imiquimod 5% cream for the treatment of her left labia majora MIS. Although there is a high recurrence rate of vulvovaginal melanomas,^1^ we postulate that the patient had 2 primary melanoma in situ lesions of the vulva based on her history and negative scouting biopsies. To our knowledge, there are only 2 other reported cases in the literature that describe multiple primary vulvar melanomas.^2^^,^^3^ Standard of care for melanoma is surgical excision, but vulvar malignancies can be a surgical challenge. Therefore, it is key to consider the appropriateness of alternative treatments, particularly in locations with increased risk of morbidity and disfigurement. Although more data is required to propose a standardized regimen for using imiquimod for MIS regarding treatment duration, protocol, and long-term efficacy, we hope this case further highlights and contributes to evaluating the role of imiquimod in the treatment of MIS in appropriate cases.

## Case presentation

**Fig 1:**
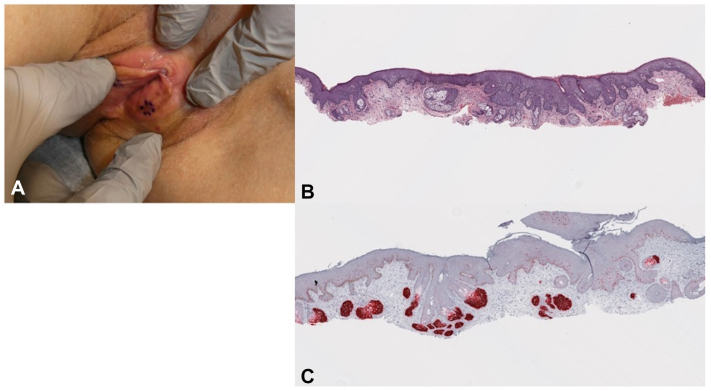
**A,** Physical exam demonstrated 2 to 4 mm pigmented macules of the left labia. *Dotted* in purple skin marker is the biopsied lesion of the left labia minora. The right labia was without evidence of any noticeable regimentation, nodularity, erosion, or ulceration. **B,** Photomicrograph of the initial biopsy (H&E, ×40). **C,** Corresponding area with the PRAME immunostaining (PRAME, ×40).

A 70-year-old female with a history of MIS of the right labia majora, biopsy-proven left labial melanosis, MIS of the left anterior shoulder, and basal cell carcinoma of the back presented to the dermatology clinic. The patient’s history of right labia majora MIS in 2018 was treated with Modified MMS; the margins were negative on histopathologic evaluation. At this encounter, the patient reported she was having difficulty monitoring a previously noted pigmented lesion in the vulva due to its location. She provided photographs of the vulva taken 4 years prior to presentation, which demonstrated comparative darkening and increased involvement of the vulva (Fig 1). Shave biopsy of the left labia minora demonstrated MIS.

**Fig 2:**
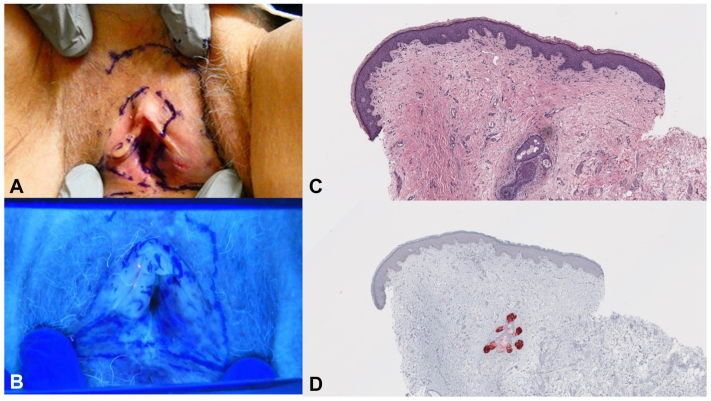
**A,** Targeted physical examination when patient returned to clinic for scouting biopsies at her second appointment. **B,** Pigmented patches highlighted with Wood’s lamp of the right and left labia minora and majora, as well as the clitoral hood. There was no evidence of pigmentation of the urethral meatus or of the vaginal canal on external examination. **C,** Photomicrograph of scouting biopsy of the left labia majora (H&E, ×40). **D,** Corresponding area demonstrating increased PRAME positive intraepidermal melanocytes. While independently the density of the melanocytes was not concerning, given the patient's recent diagnosis of melanoma in situ on the ipsilateral labia majora, the edge of an evolving melanoma in situ could not be excluded (PRAME, ×40).

The patient returned to clinic for further examination and scouting biopsies of the right labia minora, clitoral hood, and left labia majora. Locations for the scouting biopsies were selected based on subclinical involvement which highlighted under Wood’s lamp (Fig 2). The right labia minora and clitoral hood were negative for MIS. The left labia majora had increased PRAME-positive intraepidermal melanocytes, which, in the setting of a recent diagnosis of MIS on the ipsilateral side could not exclude an evolving MIS.

**Fig 3:**
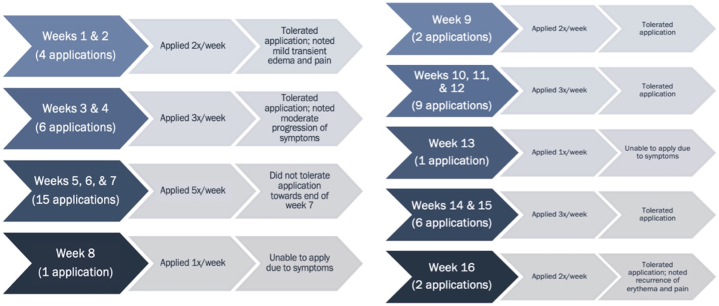
Topical imiquimod treatment application course and associated tolerability of application.

**Fig 4:**
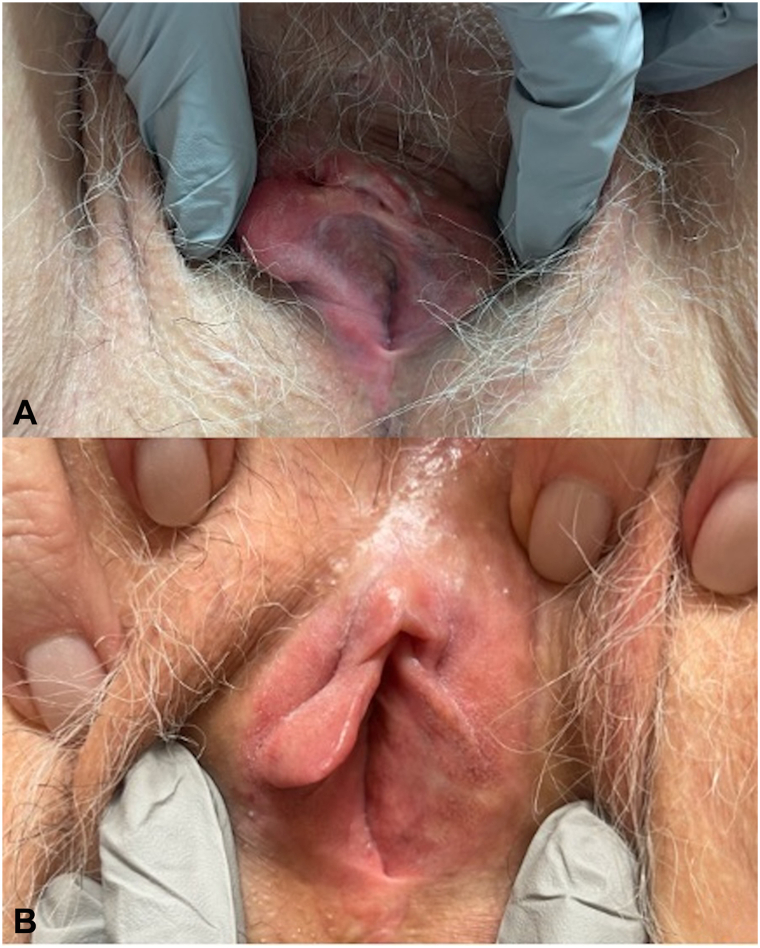
**A,** Examination after 8 weeks of imiquimod treatment demonstrated evidence of pigment regression of the left labial minora and majora, with notable erythema and edema of the surrounding vulvar skin. **B,** Examination after 16 weeks of imiquimod treatment demonstrated continued regression of pigment compared to her previous examination, with only a small, light brown macule on the left labia minora. No erythema or edema of the vulva was noted.

The patient ultimately declined surgery and elected to start off-label treatment with 5% imiquimod cream. The patient started applying imiquimod for 8-hour applications for 8 weeks before returning to clinic for reevaluation (Fig 3). After 8 weeks, there was pigment regression of the left labial minora and majora (Fig 4, *A*). She continued treatment for an additional 8 weeks (Fig 3). After 16 weeks, 1 residual pigmented macule on the left labia minora was biopsied (Fig 4, *B*). Biopsy was consistent with labial melanosis with no evidence of malignancy. The patient is continuing to undergo close follow-up.

## Discussion

While surgical excision is considered first-line for treating MIS, topical imiquimod can be utilized as a second-line therapy in cases that are not considered good surgical candidates due to age, location, or other medical comorbidities as examples.^4^ In addition to being declined by the patient given the extent of involvement, with surgery likely to be disfiguring and extensive, this represented a case where alternative treatment options to surgery were considered. A thorough discussion regarding the risks, benefits, off-label use, and uncertainties associated with nonsurgical management was conducted before proceeding with imiquimod therapy. This discussion also included the understanding that if the patient were to develop pigment progression, involvement of the urethral orifice, vaginal canal, or nodularity, we would recommend referral for surgical intervention.

Other modalities to consider for treating anatomically constrained locations include MMS and radiation therapy. MMS allows for visualization of the peripheral and deep margins and can also be utilized to preserve tissue in locations where morbidity and disfigurement are high.^4^^,^^5^ Although less commonly used, radiation therapy has been described as a nonsurgical treatment option for MIS, with the recommendation of consulting a radiation oncologist.^4^

Imiquimod is FDA-approved for the treatment of superficial basal cell carcinoma, actinic keratoses on the face and scalp, and anogenital warts.^6^ However, imiquimod has also been used off-label in the management of other disease entities. Although the mechanism of action is largely unknown, the anti-tumor effects of imiquimod are thought to be secondary to its work as a TLR-7 and TLR-8 agonist.^6^ Activation of TLR-7 and TLR-8 leads to activation of NF-κB and the production of proinflammatory cytokines.^6^ Side effects of imiquimod treatment are dose-dependent and can vary in severity, and are viewed as representing treatment effectiveness.^6^ However, the side effects may limit consistent use of imiquimod, particularly in sensitive regions. The patient was able to complete 16 weeks of treatment, but she required breaks in application due to irritation, edema, and pain. The symptoms improved with holding application and applying a low-potency topical corticosteroid. She underwent a total of 46 applications.

Although the use of imiquimod in treating MIS has been described, there is a lack of standardization regarding the approach to management. Several contributing factors include the frequency of application, length of treatment, treatment margins utilized, application under occlusion, monotherapy versus combination treatments (such as post-surgical adjuvant therapy), and the duration of follow-up monitoring for recurrence.^7^, ^8^, ^9^ It has been proposed that treatment dose and intensity impact malignancy clearance, and at least 60 applications results in a higher propensity for clearance.^5^

The treatment goal for our patient was to experience some irritation to ensure treatment effectiveness, without experiencing intolerable symptoms. The application was ideally 3 to 5 times per week for 16 weeks to meet the cumulative dose goal. Additionally, the treatment endpoint was clinical pigment resolution, followed by repeat biopsy of any residual pigment. The patient initially declined the recommended repeat scouting biopsies at her 16-week follow-up, but later agreed to performing the biopsies to confirm histopathologic clearance 9 months after treatment completion. The scouting biopsies after treatment with imiquimod were negative for any residual or recurring malignancy. To date, there has been no evidence of recurrence 13 months after completing the treatment course.

## Conflicts of interest

None disclosed.
